# Current Trends of HIV Infection in the Russian Federation

**DOI:** 10.3390/v15112156

**Published:** 2023-10-26

**Authors:** Daria Ogarkova, Anastasiia Antonova, Anna Kuznetsova, Ruslan Adgamov, Andrei Pochtovyi, Denis Kleimenov, Elena Tsyganova, Vladimir Gushchin, Aleksandr Gintsburg, Aleksei Mazus

**Affiliations:** 1 Federal State Budget Institution “National Research Centre for Epidemiology and Microbiology Named after Honorary Academician N.F. Gamaleya”, Ministry of Health of the Russian Federation, 123098 Moscow, Russia; dashadv1993@gmail.com (D.O.); anastaseika95@mail.ru (A.A.); bacter@yandex.ru (R.A.); a.pochtovyy@gmail.com (A.P.); mne10000let@gmail.com (D.K.); gintsburg@gamaleya.org (A.G.); 2 Moscow City Center for AIDS Prevention and Control, 105275 Moscow, Russia; tsyganovaelena@yandex.ru (E.T.); mazus@yandex.ru (A.M.); 3 Department of Virology, Lomonosov Moscow State University, 119991 Moscow, Russia; 4 Department of Infectiology and Virology, Federal State Autonomous Educational Institution of Higher Education I M Sechenov First Moscow State Medical University of the Ministry of Health of the Russian Federation (Sechenov University), 119991 Moscow, Russia

**Keywords:** HIV-1, Russia, incidence, prevalence, mortality, antiretroviral therapy

## Abstract

Russia remains one of the areas most affected by HIV in Eastern Europe and Central Asia. The aim of this study was to analyze HIV infection indicators and study trends in Russia using data from the Federal Statistic Form No. 61 “Information about HIV infection”. HIV incidence, prevalence, HIV testing and mortality rates (from 2011 to 2022), and treatment success rates (from 2016 to 2022) were analyzed. These indicators were compared across different federal districts (FDs) of Russia. The findings revealed a significant downward trend in HIV incidence, while a significant upward trend was observed for HIV prevalence. The mortality rate has stabilized since 2018. The coverage of HIV testing and antiretroviral therapy increased over time. The number of people living with HIV-1 (PLWH) with a suppressed viral load in Russia as a whole varied between 72% and 77% during the years under observation. The Siberian and Ural federal districts recorded the highest HIV incidence, while the North Caucasian FD reported the lowest. An increase in HIV testing coverage was observed across all FDs. This comprehensive evaluation of HIV infection indicators within the regional context contributes to the timely implementation of measures aimed at preventing the spread of HIV.

## 1. Introduction

Currently, HIV infection continues to pose a significant global public health challenge. According to World Health Organization (WHO) estimates, the global number of people living with HIV (PLWH) was approximately 39.0 million by the end of 2022. The transmission and spread of HIV-1 persist worldwide [1]. Since the onset of the global HIV epidemic, its impact has been felt across all sectors of society. Primarily affecting the working-age population, HIV infection diminishes labor resources, thereby influencing various economic processes in countries worldwide [2]. However, from the time of the first recorded case of HIV infection to the present, the disease has transitioned from the category of a fatal condition to a chronic and manageable one, primarily due to the development and implementation of antiretroviral drugs in medical practice. The primary aim of antiretroviral treatment (ART) is to prolong the healthy life of the patient. Furthermore, ART enables the suppression of virus transmission by inhibiting its replication in the bodies of each HIV-infected individual [1]. Despite its enormous benefits, ART remains a significant source of costs [3].

In 2014, the Joint United Nations (UN) Program on HIV/AIDS (UNAIDS) adopted the “90-90-90” (“95-95-95” since 2018) strategy, with the following aims: 90% of PLWH should be aware of their status; 90% of them should be accessing ART, and 90% of all patients receiving ART should have suppressed viral loads. The goal of this strategy is to significantly reduce the occurrence and spread of new cases of HIV infection in the world and, ultimately, eradicate AIDS by 2030 [4].

The only region in the world where the number of new HIV infections continues to rise is Eastern Europe and Central Asia (EECA) [5]. According to the European Center for Disease Prevention and Control (ECDC) and WHO reports, the Russian Federation had the highest rates of newly diagnosed HIV infections in the European region, with a rate of 40.2 per 100,000 population at the end of 2021 [6].

HIV infection in Russia emerged as a significant issue a decade later than in the United States, Western Europe, and Africa, due to the country’s relative isolation and limited international migration links. The first substantial increase in incidence, aside from isolated cases and a nosocomial outbreak in the late 1980s, occurred in 1996 and was linked to the introduction of HIV and its rapid spread among injecting drug users (IDUs) [7]. Presently, heterosexual transmission has risen, with more than 50% of new HIV infections stemming from unsafe heterosexual contacts [8]. The aging of the HIV-infected population has also been noted due to the increased life expectancy resulting from ART [9]. Previous studies on HIV infection at the regional level in Russia have primarily focused on molecular genetic analysis of HIV and its drug resistance [7,10,11,12,13,14,15].

In Russia, HIV infection is classified as a socially significant disease, prompting intensive research on the epidemiology of HIV infection [16]. In December 2020, the Government of the Russian Federation implemented a new State Strategy to counter the spread of HIV infection, aligning with the “90-90-90” strategy, aimed at curbing the spread of HIV infection in Russia by continually reducing the number of new cases of HIV-infection and the mortality from HIV/AIDS-associated diseases. The ultimate goal is to eliminate the threat of AIDS to public health by 2030 [17]. To facilitate statistical monitoring in healthcare, the Ministry of Health of the Russian Federation maintains the Federal Register of PLWH, using specifically designed forms that allow for the assessment of various epidemiological indicators of HIV/AIDS [18]. Thus, Federal Statistic Form No. 61 (“Information about HIV infection”) contains information about patients with HIV infection. This study was based on the data from this form.

The aim of this study was to analyze the key indicators of HIV infection in the Russian Federation from 2011 to 2022, including by federal districts, and to examine its trends. This marks the first comprehensive examination of the epidemiological situation of HIV infection in the Russian Federation.

## 2. Materials and Methods

### 2.1. Datasets

Two data sources were used for this study. The Federal Statistical Form No. 61 “Information on HIV infection” provided the necessary information to estimate HIV incidence, prevalence, HIV testing and mortality (from 2011 to 2022), and therapy success rates (from 2016 to 2022). This database encompasses the following annual indicators from the Russian Federation and its federal districts:

▪ number of new HIV infections;
▪ number of PLWH registered at the end of the year;
▪ number of HIV-infected patients removed from the register due to death;
▪ number of persons tested for HIV-1;
▪ number of PLWH receiving ART;
▪ number of patients with suppressed viral load (VL).

The website of the Federal State Statistics Service of the Russian Federation provided data on the population of Russia as a whole and by federal district [19].

### 2.2. Statistical Analyses

The analyzed intensive indicators were calculated using the following statistical formulas:

(1) $$The\;incidence=\frac{Number\;of\;new\;HIV\;infections}{Population}\cdot 100,000$$

(2) $$The\;prevalence=\frac{Number\;of\;PLWH\;registered\;at\;the\;end\;of\;the\;year}{Population}\cdot 100,000$$

(3) $$The\;mortality=\frac{Number\;of\;HIV-infected\;patients\;removed\;from\;the\;register\;due\;to\;death}{Population}\cdot 100,000$$

(4) $$HIV\;testing=\frac{Number\;of\;persons\;tested\;for\;HIV-1}{Population}\cdot 100$$

Indicators of therapy success were calculated using the following statistical formulas:

(5) $$The\;proportion\;of\;PLWH\;receiving\;ART=\frac{Number\;of\;PLWH\;receiving\;ART}{Number\;of\;PLWH\;registered\;at\;the\;end\;of\;the\;year}\cdot 100$$

(6) $$The\;proportion\;of\;patients\;on\;ART\;with\;supressed\;VL=\frac{Number\;of\;patients\;with\;supressed\;VL}{Number\;of\;PLWH\;receiving\;ART}\cdot 100$$

To evaluate 95% confidence intervals, binomial distribution was used as

(7) $$I\pm 1.96\sqrt{\frac{I\cdot (100,000-I)}{Population},}$$

where *I*—an intensive indicator expressed in persons per 100,000 population.

In the second part of the study, the above indicators were compared in different federal districts of the Russian Federation.

The trend of the long-term dynamics of incidence, prevalence, and mortality was determined using the method of least squares [20]. The alignment of time series was carried out according to the function:

(8) $$y_{1}=a+bx,$$

where $y_{1}$—rectilinear trend indicator; a—a constant value characterizing the long-term incidence (prevalence or mortality) rate; b—a variable value for each analyzed year, which forms the angle of the trend; x—analyzed time intervals.

Statistical significance was evaluated using the F-criterion and the program SPSS Statistics ver. 26 (IBM, Armonk, NY, USA). The severity of the trend was considered according to the function:

(9) $$T=\frac{b\cdot K}{a}\cdot 100\%,$$

where K=1 for an odd number of years of observation; K=2 for an even number of years of observation

If |T| >= 5%, the trend was evaluated as pronounced; if 1% <= |T| < 5%, then trend was moderate, and if |T| < 1%, the intensive indicator was stable.

To assess differences in the age of HIV-infected individuals in 2011 and 2022, the Kruskal–Wallis criterion from the scipy.stats library in Python was applied.

## 3. Results

### 3.1. Current HIV Infection Trends in the Russian Federation

**Figure 1:**
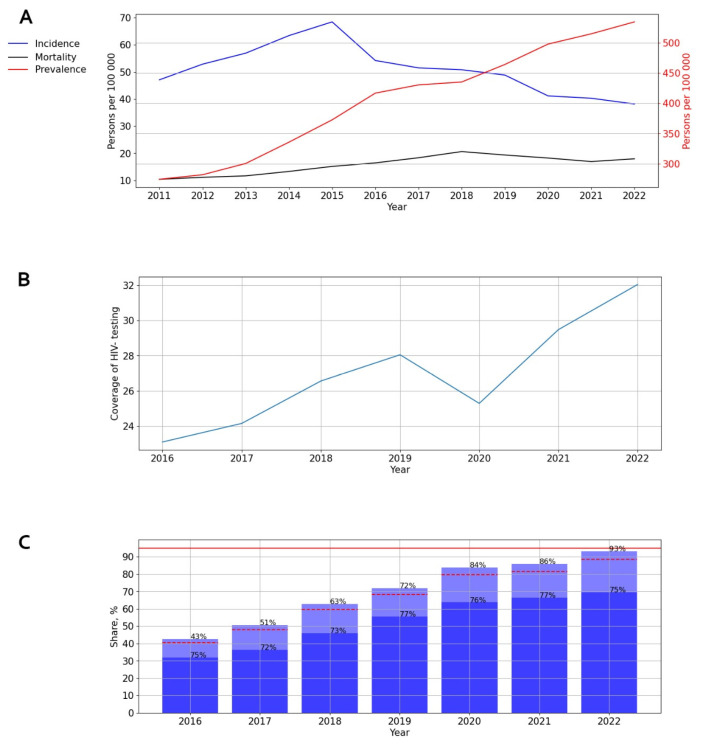
Intensive indicators of HIV infection in the Russian Federation. (**A**) Indicators of incidence, prevalence, and mortality. The left y-axis is relevant for incidence and mortality, and the right y-axis is relevant for prevalence. (**B**) Indicator of HIV testing. (**C**) Indicators of ART. Stacked column chart: the proportion of the PLWH receiving ART and the proportion of patients with suppressed VL. The red lines indicate 95% limits according to the “95-95-95” strategy.

The long-term dynamics of HIV infection incidence and prevalence and PLWH mortality in Russia from 2011 to 2022 are shown below (Figure 1A).

The incidence graph highlights two distinct periods: a slight increase in incidence rates from 2011 to 2015, followed by a subsequent decrease to 38.17 cases per 100,000 persons during 2015–2022. A significant (*p* = 0.029) and pronounced downward trend was identified, with an average annual growth rate (AAGR) of −5.61%.

When analyzing the prevalence of HIV-1 in Russia, a significant (*p* = 0.001) and pronounced upward trend was discovered, with an average annual growth rate of 10.96%.

On the mortality graph, two distinct periods can be observed: a significant (*p* < 0.001) increase in the death rate of PLWH from 2011 to 2018, followed by stabilization from 2018 onwards, with rates recorded at 19.37 cases per 100,000 persons in 2019, 18.26 in 2020, 16.95 in 2021, and 17.98 in 2022.

The coverage of HIV testing in Russia increased over time, from 21.8% in 2016 to 32.2% in 2022 (*p* < 0.001). Although a visual assessment of testing coverage revealed a decline to 24.6% in 2020, this did not impact the overall upward trend (Figure 1B).

ART coverage in Russia experienced a steady increase from 43% in 2016 to 93% in 2022. The number of PLWH with a suppressed viral load fluctuated between 72% and 77% during different years of observation (Figure 1C).

### 3.2. HIV Infection in Different Federal Districts of Russia

**Figure 2:**
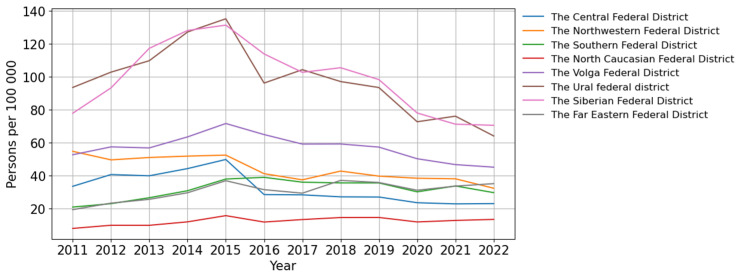
Incidence of HIV infection in the federal districts of the Russian Federation.

The Russian Federation, encompassing eight federal districts (Figure S1), holds one of the largest territories globally. The Siberian and Ural Federal Districts recorded the highest incidence of HIV infection, while the North Caucasian Federal District reported the lowest incidence (Figure 2).

A significant trend toward a decrease in the incidence of HIV was revealed in three federal districts of the Russian Federation: the Ural (*p* = 0.018; AAGR = −4.92%, moderate), the Northwestern (*p* < 0.001; AAGR = −7.66%, pronounced) and the Central (*p* = 0.005; AAGR = −11.01%, pronounced). In two federal districts, the North Caucasian and the Far Eastern, a significant and pronounced (*p* = 0.023, AAGR = 7.64% and *p* = 0.005, AAGR = 8.14% respectively) upward trend was revealed.

A visual assessment of HIV-1 incidence in the Siberian and the Volga Federal Districts demonstrated a start of decline that commenced in 2015 (Figure 2).

The Southern Federal District was characterized by an uneven distribution of incidence with periods of fluctuations (Figure 2).

**Figure 3:**
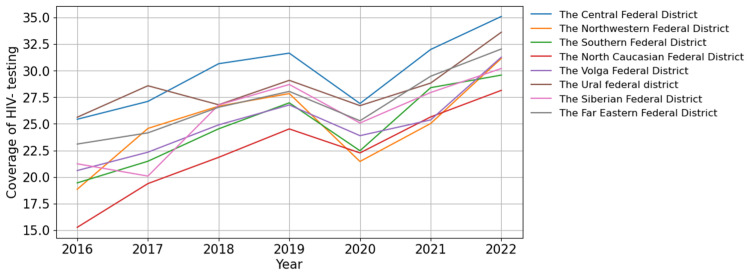
Coverage of HIV testing in the federal districts of the Russian Federation.

An increase in HIV testing coverage was observed across all federal districts of the Russian Federation, with a single decline in 2020. The Central Federal District achieved the maximum HIV testing coverage, while the North Caucasus Federal District reported the minimum (Figure 3).

#### 3.2.1. HIV Infection in the Central Federal District

**Figure 4:**
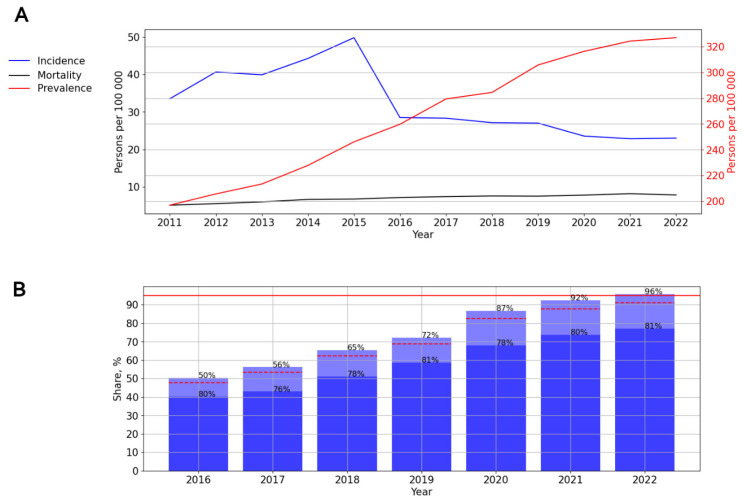
HIV infection in the Central Federal District. (**A**) Intensive indicators of the epidemic process of HIV infection. The left y-axis is relevant for incidence and mortality, and the right y-axis is relevant for prevalence. (**B**) Antiretroviral therapy. Stacked column chart: the proportion of the PLWH receiving ART and the proportion of patients with suppressed VL. The red lines indicate 95% limits according to the “95-95-95” strategy.

The long-term trends in the incidence of HIV-1 in the Central Federal District mirror those of Russia as a whole. The incidence graph illustrates two distinct periods: an initial phase of increase (2011–2015), with the incidence rising from 33.53 to 49.82 cases per 100,000 persons, followed by a limited decline continuing until 2022 (23.01 cases per 100,000 persons). A significant (*p* = 0.005) and pronounced downward trend was identified, with an average annual growth rate of −11.01% (Figure 4A).

The prevalence of HIV showed a significant (*p* < 0.001) pronounced upward trend (AAGR = 9.09%). During the study period, the mortality of people living with HIV (PLWH) also increased significantly (*p* < 0.001) from 5.11 to 7.82 cases per 100,000 persons, with an AAGR of 7.35% (Figure 4A).

The ART coverage increased over time from 50% in 2016 to 96% in 2022 (*p* < 0.001). The number of PLWH with a suppressed viral load fluctuated between 76% and 81% during different years of observation (Figure 4B).

#### 3.2.2. HIV Infection in the Northwestern Federal District

**Figure 5:**
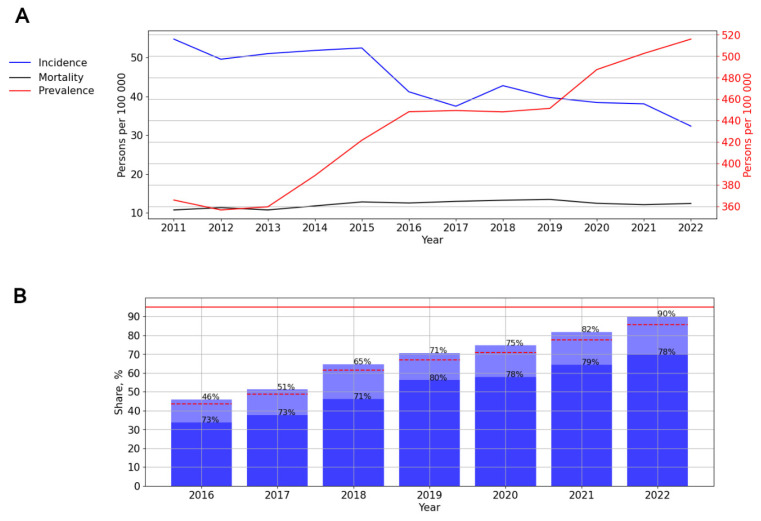
HIV infection in the Northwestern Federal District. (**A**) Intensive indicators of the epidemic process of HIV infection. The left y-axis is relevant for incidence and mortality, and the right y-axis is relevant for prevalence. (**B**) Antiretroviral therapy. Stacked column chart: the proportion of the PLWH receiving ART and the proportion of patients with suppressed VL. The red lines indicate 95% limits according to the “95-95-95” strategy.

A significant, pronounced (*p* = 0.001; AAGR = 7.77%) trend toward a decrease in the incidence was revealed. At the same time, a visual assessment of the graph showed an uneven distribution of incidence, with minor increases in 2015 (52.48 cases per 100,000 persons) and 2018 (42.78 cases per 100,000 persons) and a sharp decline in 2016 (41.19 cases per 100,000 persons) (Figure 5A).

The prevalence (*p* < 0.001) and mortality (*p* = 0.02) rates showed a significant upward trend, with average growth rates of 5.88% (pronounced) and 3.96% (moderate), respectively (Figure 5A). The ART coverage increased over time from 46% in 2016 to 90% in 2022. The number of PLWH with a suppressed viral load fluctuated between 71 and 80% during different years of observation (Figure 5B).

#### 3.2.3. HIV Infection in the Ural Federal District

**Figure 6:**
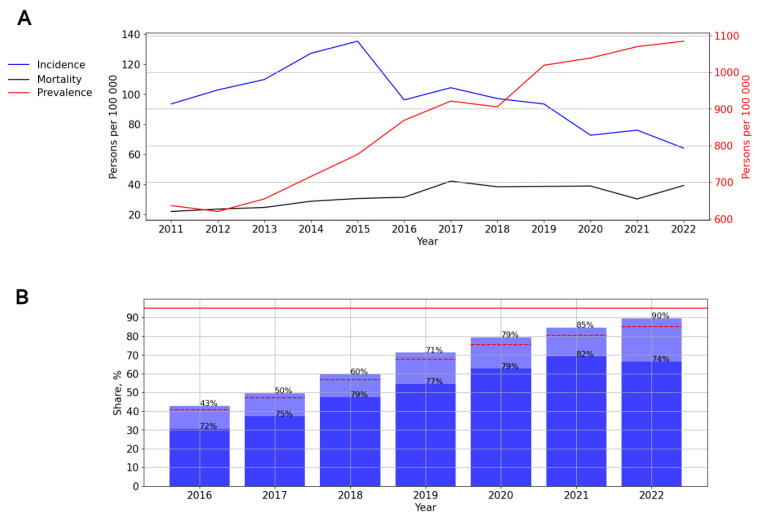
HIV infection in the Ural Federal District. (**A**) Intensive indicators of the epidemic process of HIV infection. The left y-axis is relevant for incidence and mortality, and the right y-axis is relevant for prevalence. (**B**) Antiretroviral therapy. Stacked column chart: the proportion of the PLWH receiving ART and the proportion of patients with suppressed VL. The red lines indicate 95% limits according to the “95-95-95” strategy.

A significant moderate (*p* = 0.018; AAGR = 4.92%) trend toward a decrease in the incidence was observed. In 2015, the incidence of HIV in this federal district reached a peak—135.32 cases per 100,000 persons (Figure 6A).

The prevalence (*p* < 0.001; AAGR = 10.29%) and mortality (*p* = 0.002; AAGR = 12.00%) rates demonstrated a significant, pronounced upward trend (Figure 6A).

The ART coverage increased over time, from 43% in 2016 to 90% in 2022. The number of PLWH with a suppressed viral load varied from 72% to 82% in different years of observation, with a decrease in rates in 2022 (Figure 6B).

#### 3.2.4. HIV Infection in the Volga Federal District

**Figure 7:**
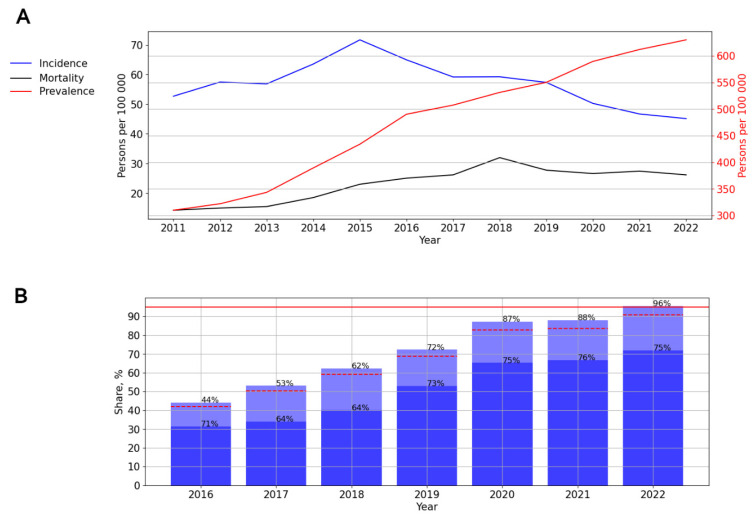
HIV infection in the Volga Federal District. (**A**) Intensive indicators of the epidemic process of HIV infection. The left y-axis is relevant for incidence and mortality, and the right y-axis is relevant for prevalence. (**B**) Antiretroviral therapy. Stacked column chart: the proportion of the PLWH receiving ART and the proportion of patients with suppressed VL. The red lines indicate 95% limits according to the “95-95-95” strategy.

Two periods can be distinguished on the incidence graph: from 2011 to 2015—A significant, pronounced (*p* = 0.016, AAGR = 7.28%) trend toward an increase, and from 2015 to 2022—A significant, pronounced (*p* <0.001; AAGR = −10.60%) trend toward a decrease in the incidence (Figure 7A).

The prevalence (*p* < 0.001; AAGR = 12.10%) and mortality (*p* < 0.001; AAGR = 14.12%) rates demonstrated a significant, pronounced upward trend (Figure 7A).

ART coverage increased over time, from 44% in 2016 to 96% in 2022. The number of PLWH with a suppressed viral load varied from 64% to 76% in different years of observation, while the lowest rates were noted in 2017 and 2018 (Figure 7B).

#### 3.2.5. HIV Infection in the Siberian Federal District

**Figure 8:**
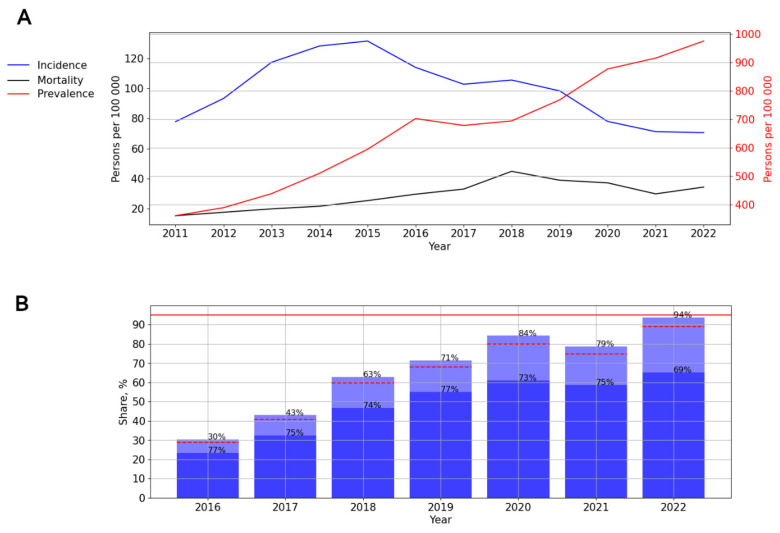
HIV infection in the Siberian Federal District. (**A**) Intensive indicators of the epidemic process of HIV infection. The left y-axis is relevant for incidence and mortality, and the right y-axis is relevant for prevalence. (**B**) Antiretroviral therapy. Stacked column chart: the proportion of the PLWH receiving ART and the proportion of patients with suppressed VL. The red lines indicate 95% limits according to the “95-95-95” strategy.

The graph of HIV incidence allows a distinction between two time periods: 2011–2015 and 2015–2022. Thus, from 2011 to 2015, a significant, pronounced (*p* = 0.007; AAGR = 12.95%) upward trend from 77.91 to 131.46 cases per 100,000 persons was observed; then, a significant, pronounced (*p* = 0.001; AAGR = −14.79%) trend toward a decrease from 102.27 (2017) to 70.60 cases (2022) per 100,000 persons was observed (Figure 8A).

The prevalence (*p* < 0.001; AAGR = 14.65%) and mortality (*p* = 0.001; AAGR = 18.55%) rates demonstrated a significant, pronounced upward trend. At the same time, during a visual assessment, two periods were distinguished: from 2011 to 2018, with an increase in mortality (*p* < 0.001; AAGR = 24.04%), and a further decline (*p* = 0.315) (Figure 8A).

ART coverage increased over time, from 30% in 2016 to 94% in 2022, while a one-time decrease in this indicator in 2021 (74%) was observed. The number of PLWH with a suppressed viral load varied from 69% to 77% in different years (Figure 8B).

#### 3.2.6. HIV Infection in the Southern Federal District

**Figure 9:**
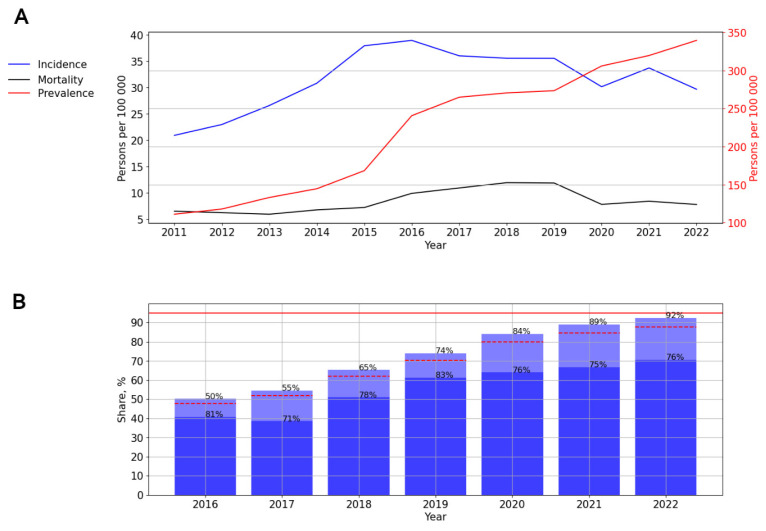
HIV infection in the Southern Federal District. (**A**) Intensive indicators of the epidemic process of HIV infection. The left y-axis is relevant for incidence and mortality, and the right y-axis is relevant for prevalence. (**B**) Antiretroviral therapy. Stacked column chart: the proportion of the PLWH receiving ART and the proportion of patients with suppressed VL. The red lines indicate 95% limits according to the “95-95-95” strategy.

The graph of HIV incidence demonstrated a significant, pronounced (*p* < 0.001; AAGR = 21.25%) trend toward an increase: from 20.91 cases per 100,000 persons in 2011 to 38.97 cases per 100,000 persons in 2016, followed by a constant decrease in incidence (Figure 9A).

The prevalence rates demonstrated a significant, pronounced (*p* < 0.001; AAGR = 18.81%) upward trend (Figure 9A).

The graph of HIV mortality demonstrated a significant, pronounced (*p* = 0.002; AAGR = 18.07%) trend toward an increase: from 6.49 cases per 100,000 persons in 2011 to 11.92 cases per 100,000 persons in 2018. Since 2018, mortality among PLWH has decreased (Figure 9A).

ART coverage increased over time, from 50% in 2016 to 92% in 2022. The number of PLWH with a suppressed viral load varied from 71% to 83% in different years of observation, with a decrease in rates from 2020. The lowest rate was noted in 2017—71% (Figure 9B).

#### 3.2.7. HIV Infection in the Far Eastern Federal District

**Figure 10:**
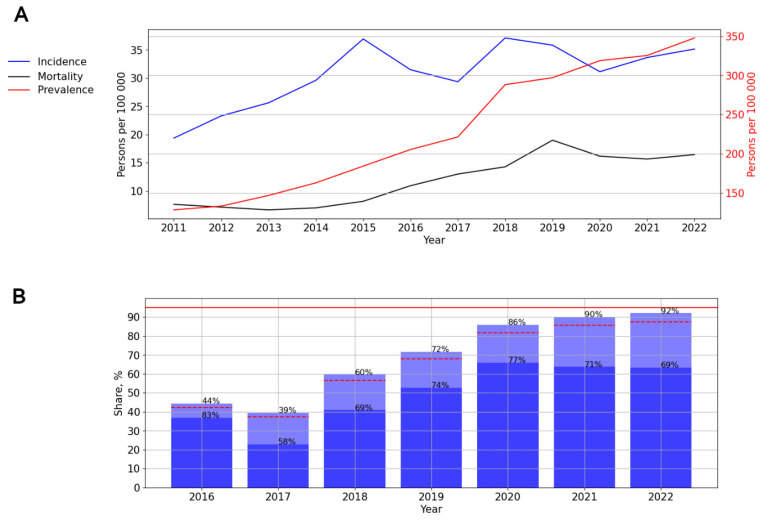
HIV infection in the Far Eastern Federal District. (**A**) Intensive indicators of the epidemic process of HIV infection. The left y-axis is relevant for incidence and mortality, and the right y-axis is relevant for prevalence. (**B**) Antiretroviral therapy. Stacked column chart: the proportion of the PLWH receiving ART and the proportion of patients with suppressed VL. The red lines indicate 95% limits according to the “95-95-95” strategy.

In this federal district, a significant, pronounced (*p* = 0.005; AAGR = 8.14%, respectively) upward trend of HIV incidence was revealed. A visual assessment of HIV incidence showed periods of rise (2011–2015, 2017–2018, and 2020–2022) and decline (2015–2017, 2018–2022) (Figure 10A).

The prevalence rates demonstrated a significant, pronounced (*p* < 0.001; AAGR = 17.18%) upward trend from 128.23 to 347.83 cases per 100,000 persons. The mortality rates also indicated a significant, pronounced (*p* < 0.001; AAGR =19.64) upward trend, with a one-time rise in 2019—18.99 cases per 100,000 persons (Figure 10A).

ART coverage increased from 44% in 2016 to 92% in 2022, with a one-time decline in 2017 (39%). The number of PLWH with a suppressed viral load varied from 58% to 77% in different years of observation. The lowest rate was noted in 2017—58% (Figure 10B).

#### 3.2.8. HIV Infection in the North Caucasian Federal District

**Figure 11:**
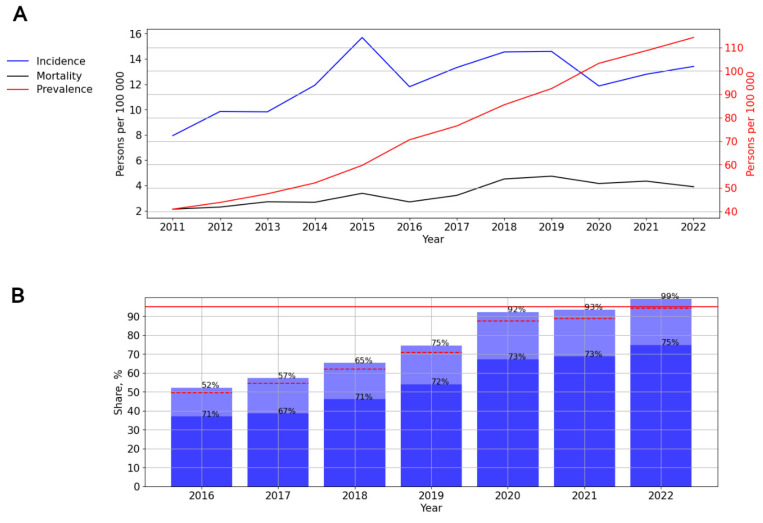
HIV infection in the North Caucasian Federal District. (**A**) Intensive indicators of the epidemic process of HIV infection. The left y-axis is relevant for incidence and mortality, and the right y-axis is relevant for prevalence. (**B**) Antiretroviral therapy. Stacked column chart: the proportion of the PLWH receiving ART and the proportion of patients with suppressed VL. The red lines indicate 95% limits according to the “95-95-95” strategy.

A significant, pronounced (*p* = 0.029; AAGR = 7.64%) upward trend of HIV incidence was revealed. In 2015, the incidence of HIV in this federal district reached a peak—15.7 cases per 100,000 persons (Figure 11A).

The prevalence rates demonstrated a significant, pronounced (*p* < 0.001; AAGR = 16.57) upward trend from 40.96 to 114.30 cases per 100,000 persons. The mortality rates also indicated a significant, pronounced (*p* < 0.001; AAGR = 16.57) upward trend from 2.13 to 3.91 cases per 100,000 persons. A visual assessment showed a stabilization of mortality rates since 2019 (Figure 11A).

ART coverage increased from 52% in 2016 to 99% in 2022. The number of PLWH with a suppressed viral load varied from 67% to 75% in different years of observation (Figure 11B).

### 3.3. Trend of Long-Term Dynamics of HIV Infection in the Russian Federation

**Figure 12:**
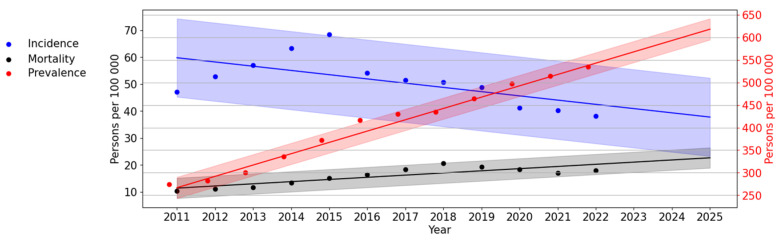
Trend lines of long-term dynamics of HIV infection in Russia. The scale on the left (black) reflects the values for the incidence and mortality trend line. The scale on the left (red) reflects values for the prevalence of HIV-1.

Trend lines of long-term dynamics of HIV infection incidence and prevalence and PLWH mortality in Russia from 2011 to 2025 are shown below (Figure 12).

An assessment of HIV theoretical incidence revealed a significant, pronounced trend towards its decrease (*p* < 0.001; AAGR = −6.43%). The difference between the theoretical indicators of the first and last years was 21.52 per 100,000 persons (or 36.01%).

An assessment of HIV theoretical prevalence revealed a significant, pronounced (*p* < 0.001; AAGR = 11.05%) upward trend. According to the trend line, HIV prevalence in Russia by 2025 will increase by 604.59 per 100,000 persons.

The PLWH mortality trend line showed a significant (*p* < 0.001) upward trend. The average annual growth rate was 9.15%.

## 4. Discussion

The epidemiology of HIV infection in Russia possesses distinct characteristics. The Russian Federation, one of the world’s largest countries, comprises eight federal districts, with significant variations in socioeconomic and demographic indicators [21]. These differences impact the spread and treatment of HIV infection.

Furthermore, there are unique aspects to the ways HIV infection spreads in Russia. The global HIV epidemic within the Russian Federation, starting in the mid-1990s, primarily affected IDUs [8]. While new cases of HIV infection have been associated with heterosexual transmission in the second half of the 2010s, the parenteral route remains a significant mode of transmission [8]. This presents challenges in HIV treatment, particularly in maintaining adherence among PLWH from the cohort of IDUs [22].

Currently, various researchers are publishing discrete data on HIV prevalence in Russia based on different sources. This study represents the first comprehensive and large-scale analysis of current trends in HIV infection in Russia, encompassing both epidemiological indicators and measures of therapy success. These indicators were analyzed in all federal districts of the Russian Federation.

The findings of this study demonstrated a significant and consistent decrease in the number of new HIV infection cases in Russia from 2015 to the present, aligning with previous research findings [23,24]. Additionally, a notable increase in the coverage of HIV testing was observed throughout the observation period (2011–2022). There was a single dip in testing coverage in 2020, likely associated with the onset of the COVID-19 pandemic.

The Siberian and Ural Federal Districts have reported the highest incidence of HIV infection, with a historical background explaining this phenomenon. During the late 1990s, a significant number of (IDUs) emerged in these regions. The Perm Territory, which was part of the Ural Federal District until 2000 and since 2000 has been part of the Volga Federal District, and the Irkutsk Region in the Siberian Federal District were among the first regions within the Russian Federation to register cases of HIV infection among IDUs [7,25,26]. This initial development set the stage for the current challenging situation regarding HIV-1 incidence in Siberia and the Urals. Previous research has also highlighted the highest mortality and HIV prevalence rates in the Siberian and Ural Federal Districts [27]. Notably, a decrease in HIV incidence has been observed in these federal districts since 2015. Simultaneously, all federal districts in Russia have continued to experience an upward trend in HIV prevalence, which can be associated with the increased life expectancy of PLWH. The median age of PLWH rose from 31 in 2011 to 41 in 2022 (*p* < 0.001) (refer to Figure S2).

The findings of this study also indicate a decline in the mortality rate since 2018. Additionally, there is an observed “aging” of the HIV-infected population, with an increase in the age of individuals who have died of HIV infection (*p* < 0.001) (Figures S2 and S3).

A decrease in the incidence of HIV was observed in all federal districts, except for the Far East and North Caucasus Federal Districts. The Far East Federal District, due to its remote location from the primary territories of Russia, exhibits unique socio-demographic characteristics. A study on migration and demographic patterns in the Russian Far East highlighted a consistent population decline, primarily attributed to migration outflow. The Far East’s share of the total Russian population decreased from 5.4% in 1991 to 4.3% in 2020, with 3.8% of its total population leaving the region in 2020 [28]. This population decrease may be correlated with the observed increase in HIV incidence in this federal district.

Conversely, the North Caucasian region is characterized by the prevalence of traditional values compared to other federal districts, leading to stigma surrounding HIV and inhibiting openness among HIV-infected individuals about their status and timely medical registration. Previous studies have noted the North Caucasian Federal District’s lowest HIV testing rates (19.4% of district residents in 2017) [29]. These findings suggest a natural increase in HIV incidence in this region.

The Southern Federal District exhibited fluctuating incidence rates, likely influenced by its high migration appeal and tourist influx.

Since 2017, the Ministry of Health of the Russian Federation has recommended administering antiretroviral therapy to all PLWH, irrespective of CD4 cell count and viral-load levels [30].

Previous reports have indicated a significant increase in ART coverage in Russia from 4% in 2006 to 58% in 2021 [31]. The development and introduction of Russian-produced antiretroviral drugs have facilitated broader access to ART for PLWH in the country, contributing to increased life expectancy. To date, 40 international non-proprietary names of drugs for the treatment of HIV infection have been registered in Russia. For the possibility of providing ART to all PLWH in Russia, antiretroviral drugs produced in Russia have been developed and actively introduced into clinical practice [32]. Since 2020, elsulfavirin, a non-nucleoside reverse transcriptase inhibitor developed in Russia, has been included in preferred first-line ART regimens in Russia [30,33].

As a result, in all federal districts, the share of PLWH receiving ART substantially increased between 2016 and 2022. Notably, in the Central, Volga, and North Caucasian Federal Districts in 2022, the share of PLWH receiving ART was more than 95%. In the Northwestern, Ural, Siberian, Far Eastern, and Southern Federal Districts in 2022, the share of PLWH receiving ART was between 90 and 94%. Generally, in Russia, the share of PLWH receiving ART increased from 43% in 2016 to 93% in 2022. This widespread availability of antiretroviral therapy has contributed to extending the lives of PLWH, resulting in an increase in the prevalence of HIV infection and the stabilization of mortality rates among HIV-infected patients by the end of the observation period.

Throughout the observation period, the proportion of PLWH with suppressed viral loads in Russia ranged from 72% to 77%. In 2022, the highest percentages of PLWH with suppressed viral loads were recorded in the Central, Northwestern, and Southern Federal Districts, accounting for 81%, 78%, and 76%, respectively. In the Ural, Volga, and North Caucasian Federal Districts, this proportion ranged from 74% to 75%. Conversely, the Siberian and Far Eastern Federal Districts reported the lowest proportions of PLWH with suppressed viral loads in 2022, at 69%. Given the significant proportion of PLWH with a history of injecting drugs in Russia, ensuring adherence to treatment remains a pertinent issue. To address this, long-acting injection therapies with bimonthly injections have been introduced in Europe, offering promising solutions for improving adherence [34]. In Russia, long-acting injectable drugs have recently been registered: Recambis (Reg. No. LP-No. (001678)-(RG-RU) from 01.17.2023) and Vocabria (Reg. No. LP-No. (001504)-(RG-RU) from 05.12.2022). Furthermore, there is ongoing development of long-acting injectable drugs within Russia, indicating progress in this domain [32].

The identified trends in the long-term dynamics of HIV-1 in the Russian Federation until 2025 indicate a decrease in the incidence of HIV with an increase in prevalence and mortality. The obtained predictive trend lines correspond to these indicators of HIV infection at its present stage in the Russian Federation and can be explained as follows: the use of highly effective ART leads to an increase in the life expectancy of PLWH, contributing to a rise in the prevalence of HIV. At the same time, the aging of the HIV-infected population over time results in a natural process of aging and death. It is also worth noting that the Federal Statistic Form No. 61 does not consider the causes of death of PLWH, implying that it might result from various factors other than HIV infection and AIDS, such as chronic diseases and accidents.

Such a comprehensive study of HIV infection indicators in the Russian Federation within a regional context not only helps in assessing the overall situation in the country but also aids in identifying regions that have a less favorable epidemiological situation. This identification enables the timely implementation of epidemiological measures and organizational arrangements to prevent the spread of HIV.

## Data Availability

The data presented in this study are available on request from the corresponding author. The data are not publicly available due to the privacy policy of the Federal Statistic Form No. 61 (“Information about HIV infection”).

## Supplementary Materials

The following supporting information can be downloaded at: https://www.mdpi.com/article/10.3390/v15112156/s1, Figure S1: Federal districts of the Russian Federation; Figure S2: Median age of PLWH from 2011 to 2022; Figure S3: Median age at death with HIV infection from 2011 to 2022.
