# Supplementary figures and images for: Current Trends of HIV Infection in the Russian Federation
Source: Viruses. 2023 Oct 26;15(11):2156. doi: 10.3390/v15112156 (PMC10674383; doi:10.3390/v15112156)

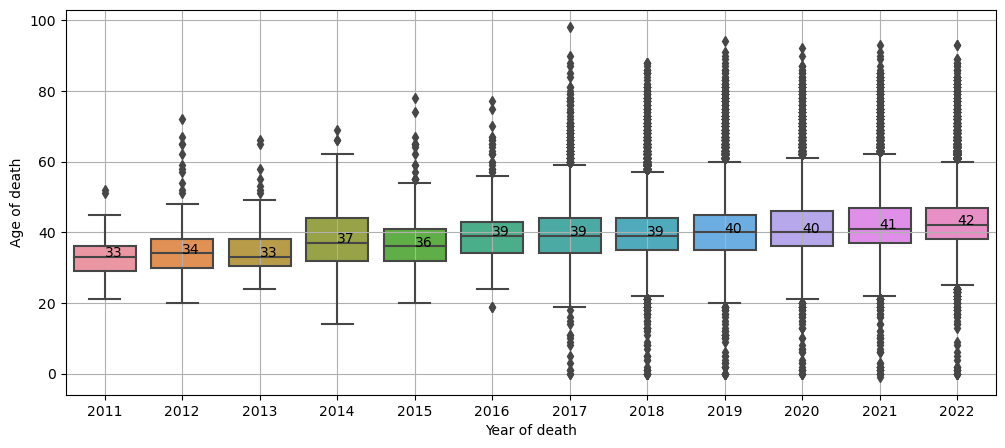

Supplement: Supplementary file 1 [file viruses-15-02156-s001.zip › FigureS3.tif]

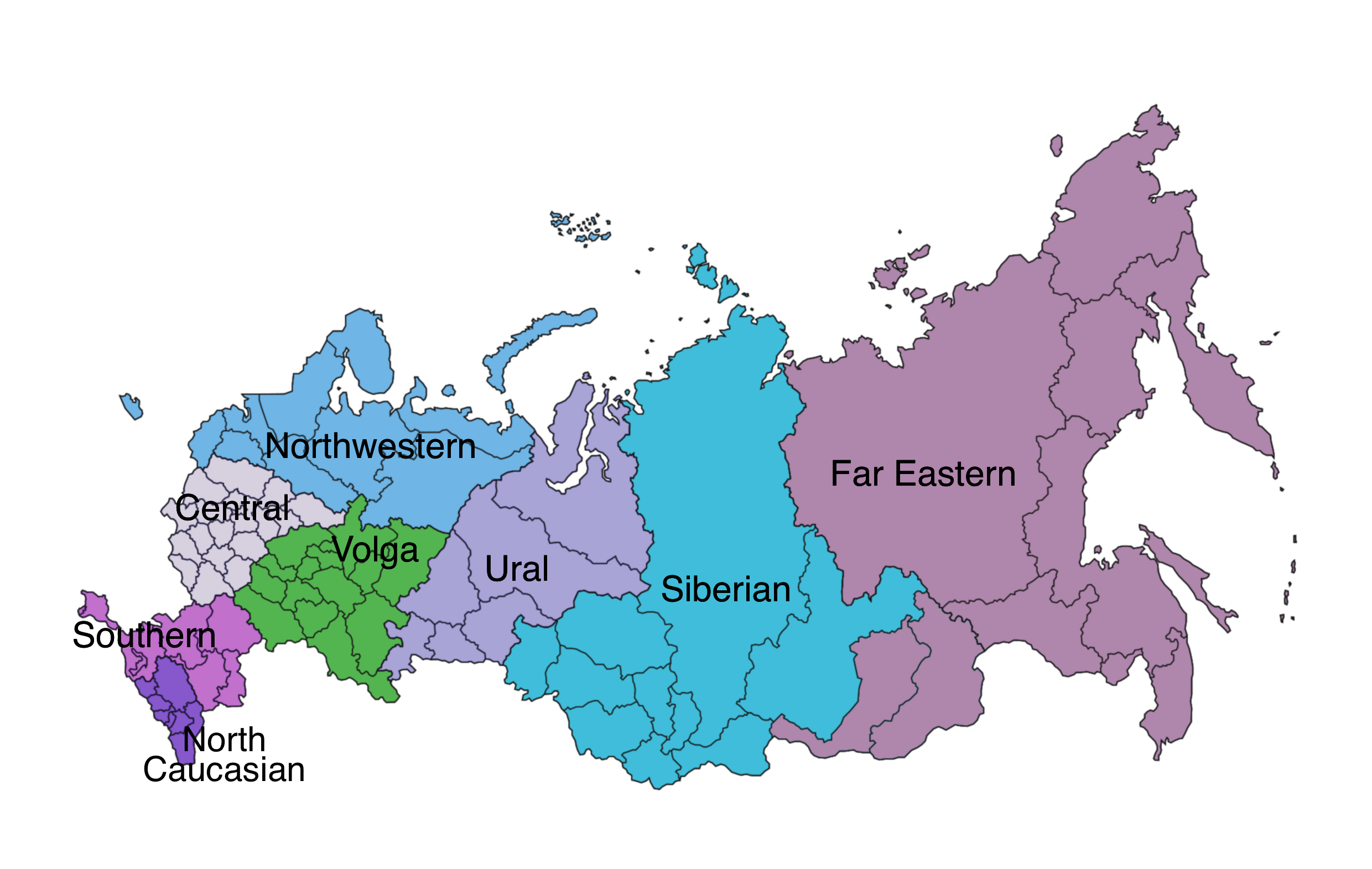

Supplement: Supplementary file 1 [file viruses-15-02156-s001.zip › FigureS1.TIF]

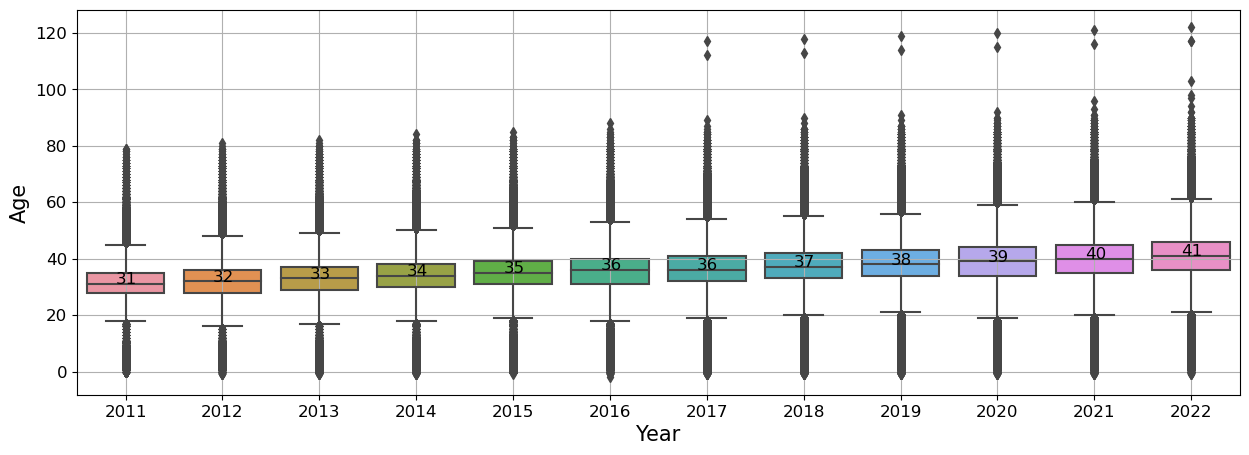

Supplement: Supplementary file 1 [file viruses-15-02156-s001.zip › FigureS2.tif]
